# Cheoplasty

**Published:** 1858-06

**Authors:** S. D. Muse

**Affiliations:** Monticello, Miss.


### CHEOPLASTY.

The following communication was received in due time, and should have been read before the Miss. Valley Association of Dentists at its last meeting in this city; but it was misplaced, and was not found till after the adjournment.

As it is one of those articles that does not *spoil* by keeping awhile, we now with much pleasure give it place in the Register and hope Dr. Muse will pardon us for what might seem to be a neglect of his article. Dr. M., let us hear from you again. —ED.

---

*Gentlemen of Miss. Valley Association of Surgeon Dentists:*

Every body has heard of "Cheoplasty," and *nearly* every one (Dentist) has bought the *Right*, yet very few there be, I imagine, who has much knowledge of the composition of the metal employed, or called by that name. True,—we know some of them and *guess* at others, and are totally unable to discover, or even surmise, how it is that any *combination* of metals of the baser sort should be as free from corrosion and destructibility as *pure* gold or platina. Yet we see from the *best* authority, or as good as any, that this is true. Shall we believe it or not? I shall not decide.

As to priority of discovery, so far as the mode is concerned, I think the same was published in the periodicals a half dozen years ago. I have not time to hunt up the article, but it is there, sure. However, in that article block tin was used or recommended instead of "*Cheoplasty*;" but there *is something* in a *name*, and there is, doubtless, something in Cheoplasty—I would not vouch for its non-corrosiveness. Vouchers to that effect notwithstanding. Very respectfully,

MONTICELLO, MISS., Jan. 20, 1858. S. D. MUSE.

more even than some are ready to admit. However, it was not to extol or condemn that I commenced this article, nor do I know how it has run into this channel; but lest I should make matters worse, I will to the *point.*

Well! I, amongst others, bought the *Right.* It would—according to recommendation—serve as a base equally well for plain, as gum teeth. For *block* work—whole sets, half sets, or a single tooth. But nothing was said of a base for "Allen's continuous gum." Well! having been exceedingly annoyed with unavailing efforts to get an adjustment of a set of teeth (the mouth being exceedingly awkward in all its parts, but more especially the palate and border with which I had to do), I concluded to make an effort with Blandy's base, with entirely satisfactory results—so far, at least, as a *fit* was concerned. It now remains to explain the process, and I am done.

From your impression get first a spar and plaster model—this is the first half of your matrix. Then get a plaster model (the spar and plaster first, as it is best, and indispensable to have this perfect.) Fit your wax plate over this, and take up the impression in sand; get up your metallic model and counter, as usual—then cut your plate just a little wider (say a line) than you wish your continuous gum border. Strike up your plate, then adjust another plate, wax, same thickness as the first to the plaster model; on this lay your platina plate. Now you are ready to take up the articulating model, or get the *bite*, if you prefer this expression. This done, you adjust your teeth—remove platina from wax plate, and proceed as usual with Allen's style, until *gummed.* Then trim the edges within a line of the gum—perforate the border of the plate with a plate punch, replace on the wax plate, this time, on the spar and plaster model; build wax just where you wish the metal to flow; varnish the border of model; oil; encircle with tin ring; pour the second half of matrix; separate, prepare and pour the metal according to Blandy, and you will have a *fit sure*, and a beautiful piece of work. But

